# Effectiveness of F3 feline facial pheromone analogue for acute stress reduction within clinical veterinary practice

**DOI:** 10.18849/ve.v8i4.669

**Published:** 2023-12-06

**Authors:** Ebony Crump+

**Keywords:** BEHAVIOUR, CATS, FELINE FACIAL PHEROMONE, FELINE FRIENDLY, FELINE, FELIWAY, HANDLING, STRESS

## Abstract

**PICO question:**

In cats within a clinical veterinary context, does the application of the F3 feline facial pheromone (Feliway™), when compared to placebo, reduce signs of acute stress?

**Clinical bottom line:**

**Category of research:**

Treatment.

**Number and type of study designs reviewed:**

Five papers were critically reviewed. There were three prospective, double-blinded, randomised controlled trials, one prospective, single-blinded, randomised controlled trial and one prospective, single-blinded, non-randomised controlled trial.

**Strength of evidence:**

Moderate.

**Outcomes reported:**

Four studies found improvement in select indicators of acute stress following F3 feline facial pheromone analogue (FFPA) exposure. One study showed FFPA reduced patient stress during routine physical examination, and improved caregiver impression of patient relaxation and ease of handling. One study revealed FFPA decreased vocalisations but had no effect upon systolic blood pressure during physical examination. One study determined that FFPA calmed but did not reduce struggling during venous catheterisation. One study demonstrated reduced time to sedation and propofol induction dose for routine surgical procedures when a transport protocol incorporating FFPA was applied. Finally, one study found no significant effect of FFPA upon behavioural and physiologic measures of acute stress during physical examination. No studies reported outward negative effects associated with FFPA exposure.

**Conclusion:**

It can be concluded that FFPA may reduce signs of acute stress within a clinical veterinary context. Additionally, exposure to FFPA is unlikely to cause patient harm. To optimise patient welfare, FFPA is not recommended as a sole agent for stress mitigation and should instead be incorporated holistically with patient friendly handling, clinic design, and pharmacotherapy where indicated.

## How to apply this evidence in practice

The application of evidence into practice should take into account multiple factors, not limited to: individual clinical expertise, patient’s circumstances and owners’ values, country, location or clinic where you work, the individual case in front of you, the availability of therapies and resources.

Knowledge Summaries are a resource to help reinforce or inform decision making. They do not override the responsibility or judgement of the practitioner to do what is best for the animal in their care.

## Clinical scenario

Animals of the same species may emit and detect chemical cues capable of influencing reproductive and social behaviour, known as pheromones (Frank et al., 2010; and Vitale-Shreve & Udell, 2017). Five pheromones (F1 to F5) have been isolated from sebaceous cheek gland secretions of the domestic cat (*Felis catus*) (Mills, 2005; Pageat & Gaultier, 2003; and Vitale-Shreve & Udell, 2017). The F3 fraction of feline facial pheromone is deposited when the cheek is rubbed against objects within the environment, in a process known as ‘bunting’ (Da Silva et al., 2017; DePorter, 2016; and Pageat & Gaultier, 2003). It is associated with spatial orientation and perception of territory (Da Silva et al., 2017; and Pageat & Gaultier, 2003). As a species, cats are recognised as solitary hunters that evade and avoid threats to survive (Ellis et al., 2013; Griffith et al., 2000; and Rodan et al., 2011). Consequently, they prefer familiar environments which facilitate a sense of control (Ellis et al., 2013; and Riemer et al., 2021). As such, application of synthetic F3 feline facial pheromone analogue (FFPA) may be associated with stress reduction in cats when they are placed in unfamiliar environments by signaling that they are within safe territory (Da Silva et al., 2017; and Griffith et al., 2000).

In cats, veterinary visits are associated with acute stress due to changes in routine, loud or unfamiliar noises, unfamiliar environment, presence of strangers (humans, cats, or other animals), and physical manipulation (Amat et al., 2016; Shu & Gu, 2021; and Stella & Croney, 2019). Stress has a negative impact upon patient welfare and the immune system (Amat et al., 2016; Marti et al., 2016; and Tateo et al., 2021). Additionally, in a clinical setting the physiologic effects of stress can impede safe animal handling and confound the outcome of physical examinations and diagnostic tests (Griffith et al., 2000; and Urrutia et al., 2019). Consequently, the reduction and mitigation of stress has become a priority in clinical veterinary practice (Herron & Shreyer, 2014; Riemer et al., 2021; and Rodan et al., 2011). Besides improving the patient experience, it has been recognised that implementing low-stress methods promotes caregiver satisfaction and clinic loyalty, and reduces rates of staff injury (Anseeuw et al., 2006; Herron & Shreyer, 2014; Marti et al., 2016; and Tateo et al., 2021). The application of FFPA has been recommended as a measure to decrease stress in cats without associated adverse effects (Anseeuw et al., 2006; Da Silva et al., 2017; Griffith et al., 2000; and Shu & Gu, 2021). Consequently, this review aims to investigate such claims in association with acute stress lasting 1 hour or less within a clinical veterinary context (Dickerson & Kemeny, 2004).

## The evidence

All studies specifically investigated the effect of the F3 fraction of feline facial pheromone, hereafter referred to as FFPA. Two prospective, double-blinded, randomised controlled trials compared the effect of FFPA to placebo (Conti et al., 2017), and placebo and no treatment (Pereira et al., 2016), upon physiologic and/or behavioral parameters during clinical examination. One also investigated the effect of environment, comparing the home and a clinical context (Conti et al., 2017). A third prospective, double-blinded, randomised controlled trial compared the effects of FFPA combined with acepromazine, placebo combined with acepromazine, FFPA alone, and placebo alone, upon behavioral parameters during intravenous catheterisation (Kronen et al., 2006). One prospective, single-blinded, randomised controlled trial compared the effects of FFPA application and bypassing the waiting room upon blood pressure, physiologic, and behavioral outcomes to no treatment (Van Vertloo et al., 2021). Finally, one prospective, single-blinded, non-randomised controlled trial incorporated FFPA into a low-stress transport protocol applied before routine anaesthesia, to determine its effect upon pre-anaesthetic and behavioral parameters when compared to no treatment (Argüelles et al., 2021).

## Summary of the evidence

**Argüelles et al. (2021):** 

Population:	Recruitment: Feline patients sterilised between December 2015 and March 2018 at the Centro Veterinario Integral La Canada veterinary clinic, located in Valencia, Spain. Caregivers were offered the choice to follow a low-stress transport protocol at the preoperative appointment, which included the use of F3 feline facial pheromone analogue (FFPA) (Feliway Classic™). Cats of caregivers who followed the low-stress protocol on the day of surgery were recruited to the treatment group. Criteria for eligibility and inclusion: American Society of Anesthesiologists (ASA) class 1; young and healthy without any previous pathology. Undergoing elective sterilisation surgery. Previously visited a veterinary clinic no more than three times, and only for the purpose of routine vaccination. Transported to the veterinary clinic for a maximum duration of 10 minutes by car. Owned, informed consent and authorisation obtained from caregiver. Criteria for exclusion and rejection: Females in oestrus or pregnant. Trained to any degree to tolerate the carrier environment and / or car transport. Administered anxiolytic medication prior to transport. Enrolled study population: 32 males and 35 females (reproductive status not reported). Ages ranging from 5–36 months. Crossbreed and pure breed cats.
Sample size:	67 cats.
Intervention details:	Group allocation: Non-randomised. Treatment: Caregivers followed the transport protocol correctly. n = 31 (18 males, 13 females). Control: Transport protocol followed but FFPA not applied, or no aspects of the protocol followed. n = 36 (14 males, 22 females). Blinding: Single-blinded. Investigators were unaware of group allocation. Caregivers were aware of group allocation. Low-stress transport protocol: Carriers were either new or thoroughly cleaned with an enzymatic cleaner. Each inside corner of the carrier was sprayed with one pump of FFPA at least 30 minutes before occupancy. Cars were sprayed with three pumps of FFPA at least 30 minutes before carriers placed inside. Carriers were placed on the floor behind the front passenger seat and secured in place. Upon arrival at the clinic, a veterinary nurse transported the carrier directly to a cat specific consultation room equipped with a FFPA diffuser (Feliway Classic™). Treatment application: Patient removed from carrier and general physical examination performed; heart rate, respiratory rate, weight. Medetomidine 0.01 mg/kg, pethidine 5 mg/kg and midazolam 0.3 mg/kg intramuscular (IM) sedation administered. Patient returned to carrier and lights dimmed. After 3 minutes, patient checked for appropriate sedation, sedation was considered appropriate if the patient was in lateral or sternal recumbency and did not resist handling for intravenous (IV) catheter placement. If sedation was considered insufficient, patient was checked at 2 minute intervals until 15 minutes had elapsed, at 15 minutes, supplemental sedation was administered at the discretion of the veterinarian. After IV catheter placement, patients were clipped and scrubbed for surgery and taken to the operating room. Jugular blood sample taken for routine haematology and biochemistry, and plasma cortisol levels. Propofol 0.5 mg/kg IV boluses administered if required until endotracheal intubation achievable.
Study design:	Prospective, single-blinded, non-randomised, controlled trial.
Outcome studied:	Character of the animal (subjective): A custom designed 0–3 scale was used to score the response of the patient to human contact. 0: affectionate cat, seeks contact, can be touched and lifted off the ground. 1: friendly cat, allows contact, tries to evade being caught, is not aggressive. 2: distant cat, afraid of humans, inhibited but not aggressive. 3: irascible cat, intensely fearful, aggressive. Handling before sedation (subjective): A custom designed 0–3 scale was used to score the ease of patient handling during general physical examination and injection of IM sedation. 0: impossible to handle and carry out a general examination, double towel technique for restraint is required for IM injection. 1: general examination possible but double towel technique is required for IM injection. 2: general examination possible and low-level restraint required for IM injection (two people were needed to complete the exam and/or the IM injection). 3: general examination and IM injection performed with no restraint required (one person was able to carry out both procedures without assistance). Quality of sedation (subjective): A custom designed 0–3 scale was used to score the level of sedation achieved 15 minutes after IM injection. 0: animal awake, able to walk, needs supplemental sedation. 1: animal in sternal recumbence but uncooperative, assistance needed to place the IV catheter. 2: animal in sternal / lateral recumbence, cooperative, no assistance needed to complete work. 3: animal in lateral recumbence, deep sedation, no assistance needed to complete work. Cardiac rate and respiratory rate (objective): Measured by auscultation before and after IM injection. Sedation time (objective): The time between IM injection and patient achieving ‘quality of sedation’ level 3 with a maximum time of 15 minutes, the theoretical onset of all the three drugs used for sedation. Cortisol levels (objective): Plasma cortisol levels assayed via chemiluminescence technique. Induction dose (objective): Total propofol dose administered using 0.5 mg/kg IV boluses if required.
Main Findings (relevant to PICO question)	No negative effects were observed through the application of the low-stress transport protocol. No significant difference was found between groups in terms of age, sex, weight, cardiac rate, respiratory rate, character of the animal, handling before sedation, quality of sedation, or plasma cortisol levels. Median time to sedation was 3 minutes in the treatment group, and 6 minutes in the control group (P < 0.0001). 24/31 (77%) patients from the treatment group and 14/36 (39%) patients from the control group did not require administration of propofol for induction. Median propofol induction dose was 0.0 mg/kg in the treatment group and 0.9 mg/kg in the control group (P = 0.004).
Limitations:	The non-randomised study design may have introduced bias, factors influencing caregiver compliance with the low-stress transport protocol were not investigated and may have included practical viability, attitudes towards cat welfare, and caregiver demographics. No evidence of statistical power and sample size calculation. Distance of the nozzle for FFPA sprays was not defined. Many acutely stressful events occurred throughout the study (transport, examination, IM injection), complicating subjective behavioral interpretation. No conflict of interest declaration section provided within the paper.

**Conti et al. (2017) (also including a Letter to the Editor from Beck [2017]):** 

Population:	Recruitment: Method of recruitment was not defined. Criteria for eligibility and inclusion: Healthy based upon physical, electrocardiography, echocardiography, and full laboratory evaluation. Home environments with a maximum of two cats living together harmoniously and without changes to routine. Owned, informed consent and authorisation obtained from caregiver. Enrolled study population: 15 males and 15 females (reproductive status not reported). Mean age 3.5 ± 2.8 years. Domestic short hair 27/30 (90%) and Persian 3/30 (10%) breed cats. Mean weight 4.6 ± 0.9 kg.
Sample size:	30 cats.
Intervention details:	Group allocation: Randomised (method not defined). Evaluations were performed in each location over a period of 16 days, cats were evaluated for a total of 6 days, with intervals of 48 hours between examinations. The first location for each cat to be evaluated in was randomly allocated, then the first intervention was randomly allocated and subsequently alternated over the evaluation period. Cats were then evaluated in the second location for another 16 days, the first intervention was again randomly assigned, then alternated over the evaluation period. In total, cats were evaluated in each of the four test environments on three separate occasions. The period of each examination was not defined. Blinding: Double-blinded. Reported as double-blinded, however a description of blinding was not provided. Consultation locations:Home: A preferred room of the animal was selected for evaluation, approximately 9 m2 in size. In homes with two cats, evaluations were performed on individual cats in separate rooms Veterinary hospital: A standard clinic office was selected for evaluation, approximately 9 m2 in size. The room was cleaned thoroughly between appointments. When two cats were evaluated on the same day, two different offices with the same architecture were used. A minimum of 24 hours elapsed between introducing another study participant to the room. Interventions: Substance was applied throughout the location to every protuberant object and site with a depression 10 cm from objects and 20 cm from the floor. Treatment: F3 feline facial pheromone analogue (FFPA) (Feliway Classic™). Placebo: 70% ethanol spray. Total environments evaluated: Home with placebo (HP). Home with FFPA (HFFPA). Veterinary hospital with placebo (VHP). Veterinary hospital with FFPA (VHFFPA). Consultation procedure: Fifteen minutes after intervention application, cats were introduced to the environment, no manipulations were performed in the first 10 minutes to allow cats to acclimate. Evaluations were performed in the same order, beginning with the least stressful intervention of respiratory rate, followed by heart rate, systolic blood pressure, and electrocardiography. Stress related behaviours were monitored throughout. Caregivers confirmed that cats returned to normal activity the same day following evaluation, even when stress related behaviours were exhibited.
Study design:	Prospective, double-blinded, randomised, controlled, crossover trial.
Outcome studied:	Stress related behaviour (subjective): Observed during all aspects of evaluation, behaviours noted were struggling, vocalisation and agitated behaviour. Respiratory rate (objective): Measured through distance examination without physical manipulation. Heart rate (objective): Minimal physical restraint applied for auscultation. Systolic blood pressure (objective): Measured in right lateral recumbency using vascular Doppler with headphones. Electrocardiography (objective): Measured in right lateral recumbency using multichannel digital electrocardiography (ECG). Then values of consecutive R-R intervals analysed to evaluate vagosympathetic balance. Heart rate variability (HRV). Vasovagal tonus index (VVTI).
Main Findings (relevant to PICO question)	No significant differences in respiratory rate or heart rate occurred in association with FFPA application (P > 0.05). No significant differences in systolic blood pressure were noted in any groups on any day, HP (P = 0.9843), HFFPA (P = 0.9914), VHP (P = 0.9292) and VHFFPA (P = 0.9623). The use of FFPA did not have a significant effect upon stress related behaviour during manipulation, HRV, or VVTI (P > 0.05).
Limitations:	Due to the journal word count, some information pertaining to methodology was omitted, clarification was sought in a Letter to the Editor by Beck (2017). This document and the subsequent response by Conti et al. has been synthesised throughout this summary of evidence. Method of recruitment was undefined. Reproductive status of participants was not reported. Exclusion criteria for participants was not defined. Method of randomisation was not reported. Equivalence of age across treatment groups was not reported. Incomplete description of operator blinding, study described as double-blinded, but explanation was not provided. Handling techniques were not defined but examination procedure was kept consistent. Number of pumps of FFPA spray applied was not defined. Respiratory rate and heart rate may have been influenced by body temperature – though not reported in the original article, the authors clarified that rectal temperature measurements were recorded for every examination and no significant differences were noted in any test environment. Testing over a long period of time may have induced chronic stress in the study population however reporting from caregivers indicating that cats returned to normal activity following evaluations lessens the likelihood of this occurrence.

**Kronen et al. (2006):** 

Population:	Recruitment: Feline patients admitted for castration, ovariohysterectomy, dentistry, or declawing between February 1999 and February 2000 to the Cornell University Hospital for Animals, Ithaca, USA. Criteria for eligibility and inclusion: Healthy based upon physical, complete blood count, and biochemical evaluation. Criteria for exclusion and rejection: Presenting for a medically indicated surgical procedure. History of aggressive behaviour (not defined). Enrolled study population: 77 cats (sex and reproductive status not reported). Ages ranging from 6 months to 4.5 years.
Sample size:	77 cats.
Intervention details:	Group allocation: Randomised (block randomisation by lottery) Cats were randomly allocated into four groups. Intervention was randomly assigned to each cat by the hospital pharmacy department. Blinding: Double-blinded. All investigators were unaware of group allocation. Interventions: Substances were packaged in identical spray bottles and sprayed onto cage paper in three locations, then placed within the cage 1–1.5 minutes before the cat. Each spray was applied for 1–2 seconds, 10 cm from the paper surface. Treatment: F3 feline facial pheromone analogue (FFPA) (Feliway Classic™). Placebo: Feliway Classic™ carrier medium (undefined). Treatment groups: Acepromazine and FFPA (aceFFPA), n = 20. Acepromazine and placebo (acePlac), n = 19. FFPA only (FFPA), n = 19. Placebo only (Plac), n = 19. Test procedure: Glycopyrrolate 0.01 mg/kg and oxymorphone 0.05 mg/kg subcutaneous (SC) sedation administered. Additional sedation of acepromazine 0.04 mg/kg SC administered to two of the treatment groups, one receiving FFPA and one placebo. Cats placed in a cage within a ward with other animals present (cats and dogs) designed to simulate sights, sounds, and odours typically encountered in a busy veterinary hospital environment. Cats left for 24 minutes for sedation to take effect, then videotaped within the cage for 2-4 minutes. Cats removed from the cage and prepared in an adjacent anaesthesia induction room for intravenous (IV) catheterisation by a final-year veterinary student who had previously successfully catheterised at least one cat. 22G 25 mm over-the-needle IV catheter placed. Based upon initial response to catheterisation, additional chemical restraint of ketamine 5 mg/kg IM administered at the discretion of the veterinary student. Cages thoroughly cleaned and disinfected after each trial and an interval of at least 2 hours maintained between any two study participants.
Study design:	Prospective, double-blinded, randomised, controlled trial.
Outcome studied:	Stress related behaviour (subjective): A custom designed scale modified from a behavioural assessment tool for unmedicated cats in a shelter environment was used. The scale was reviewed by an animal behaviourist and animals were scored 1–5 in 10 categories. In each category, specific behaviours were defined and ranked numerically from 1–5. Numerically higher scores correlated with a greater degree of perceived calmness. Eyes: Pupil size, eyelid opening. Positioning: Ears, whiskers, head, tail, legs. Body position. Activity level. Position in cage. Degree of sedation (subjective): A custom designed 1–5 scale was used to score the level of sedation. Sedation was defined as a noticeable reduction in a cats’ reaction to environmental stimuli. 1: Not sedate. 2: Minimally sedate. 3: Weakly sedated. 4: Sedate. 5: Very sedated (zoned out). Impression of calmness (subjective): A custom designed 1–5 scale was used to score calmness. Calmness was defined as a qualitative modification of behavioural reactions to environmental stimuli. Cats were assumed to be calm if they took a greater interest in their surroundings and their behaviour suggested that they were more at ease. 1: Not calm (anxious, fearful, very tense). 2: Weakly calm, somewhat tense. 3: More calm than tense. 4: Calm. 5: Very calm. Response to catheterisation (subjective): After catheterisation response was scored into three categories. 1: No struggling. 2: Struggling but ketamine withheld. 3: Ketamine given.
Main Findings (relevant to PICO question)	There were no detrimental behavioural effects or anaesthetic reactions to FFPA observed. FFPA did not significantly reduce struggling in cats at the time of sedation (P ≤21). In terms of sedation, aceFFPA cats scored significantly lower than acePlac cats (P < 0.06). In terms of calmness, aceFFPA cats had significantly higher scores than acePlac cats in head position (P < 0.02) and position in the cage (P < 0.04). FFPA cats had significantly higher scores, indicating calmness, than Plac cats in body position (P < 0.06) and leg position (P < 0.06).
Limitations:	History of aggressive behaviour undefined in exclusion criteria. Sex, reproductive status, and breeds of study population not reported. Method of caregiver recruitment undefined. No evidence of statistical power and sample size calculation. Composition of the carrier medium used for placebo undefined. Handling techniques not defined. Number of pumps of FFPA spray applied not defined. Unvalidated methods for assessing stress related behaviour, degree of sedation, and impression of calmness employed. The impression of calmness scale relied upon the assumption that calm cats are more likely to be inquisitive and active within the cage and as such, a reduction in stress may also have been interpreted as a lower degree of sedation. Stress response is variable between individuals, stressed animals with a ‘flight’ or ‘fiddle’ response may have been more active within the cage. Researchers noted difficulty with determining position in the cage from the video footage, cage floor markings to distinguish the front, middle, and rear of the cage would have helped reduce ambiguity. Activity level was not defined by researchers and may have led to variation in interpretations between observers where one observer may have felt an active cat was one that moved within the cage, while another may have felt they only observed their environment actively. Partial funding provided by Abbot Laboratories (Feliway™ product distributor), making sponsorship bias possible.

**Pereira et al. (2016):** 

Population:	Recruitment: Feline patients presenting for consultation at the Veterinary Hospital of Braga, Portugal. Presentations included prophylaxis 36/87 (42%), annual check-up 20/87 (23%), preoperative assessment 3/87 (3%), brushing, nail trimming or microchip implantation 3/87 (3%) and undefined 25/87 (29%). When cats arrived at the hospital, caregivers were invited to participate in the clinical trial. Criteria for eligibility and inclusion: Any sex, neuter status, breed, indoor / outdoor lifestyle. Older than 26 weeks of age. Previously visited a veterinary hospital at least once. Owned, informed consent and authorisation obtained from caregiver. Criteria for exclusion and rejection: Previously diagnosed medical condition, or medical condition diagnosed throughout consultation. Currently receiving routine medication. Waited longer than 25 minutes for consultation. Enrolled study population: 49/87 males (56%) and 38/87 females (44%), reproductive status not reported. Mean age 3 years, ages ranging from 7 months to 15.8 years. Domestic short hair 54/87 (62%), pure breed 8/87 (9%), and undefined breed 25/87 (29%) cats.
Sample size:	87 cats.
Intervention details:	Group allocation: Randomised (simple randomisation, based upon order of arrival at the clinic). Treatment: F3 feline facial pheromone analogue (FFPA) (Feliway Classic™) applied. n = 29: 18/29 males (62%) (9/18 castrated [50%]), 11/29 females (38%) (7/11 spayed [64%]). Placebo: Placebo solution applied. n = 33: 17/33 males (52%) (7/17 castrated [41%]), 16/33 females (48%) (9/16 spayed [56%]). Control: No solution applied. n = 25: 14/25 males (56%) (5/14 castrated [36%]), 11/25 females (44%) (9/11 spayed [82%]). Blinding: Double-blinded. Investigators and caregivers were unaware of group allocation. Consultation locations: Two separate, non-communicating consultation rooms of similar size with identical internal layouts were labelled room A and B. Interventions: Solution bottles identically prepared and labelled B and L. Five pumps of the spray were applied to the examination table 15 minutes before each consultation. Treatment (L): FFPA spray. Placebo (B): alcohol-based spray. Test procedure: Preparation: during the first 15 days, the observer was familiarised with the evaluation tools and consultation procedure, data was not recorded or analysed. Control: during the next 15 days, the observer logged the behaviour of each cat in one of the two consultation rooms with no sprays being used, serving as a control sample. Interventions: during the next month, the observer logged the behaviour of each cat in one of the two rooms – spray ‘B’ was used on room A’s examination table and spray ‘L’ on room B’s table. Washout: a week of no observations followed, and no data was collected. Control: during the next 15 days the observer logged the behaviour of each cat in one of the two consultation rooms with no sprays being used, these values were combined with the first control sample. Interventions: during the last month, both sprays were used on the tables in each of the consultation rooms in reverse order – spray ‘L’ was used on room A’s examination table and spray ‘B’ on room B’s table. Consultation procedure: Consultations were performed in the assigned room and followed the same sequence of general data collection, history taking, and physical examination. After 15 minutes had elapsed, the observer entered data based upon the cats Cat Stress Score (CSS) and ease of handling at that point in time, then the owner’s opinion was added to the form. All consultations were carried out by the same veterinarian and every assessment recorded by the same observer, the owner of each cat attended the full consultation. Between consultations the examination table was cleaned with a standard surface disinfectant and paper towels, the floor was mopped, and the room was aired by opening the door.
Study design:	Prospective, double-blinded, randomised, controlled trial.
Outcome studied:	Stress level without interaction (subjective): The CSS developed by Kessler and Turner (1997) was used to score cats on a 1–7 scale based upon postural and behavioural reactions. Beginning at (1), the score includes ‘fully relaxed’, ‘weakly relaxed’, ‘weakly tense’, ‘very tense’, ‘fearful and/or stiff’, ‘very fearful’, and ‘terrified’. Ease of handling (subjective): A custom designed 1–5 scale was used to score the ease of handling. 1: cat accepts handling – cat approaches the surgeon, looks for contact with a friendly posture and handling is performed effortlessly. 2: easy to handle – cat won’t approach the surgeon but does not resist handling allowing the necessary procedures for the normal course of the consultation to be carried out. 3: reluctant to be handled – cat is agitated and tries to flee from handling, handling is still achieved with little physical restraint. 4: aggressive and difficult to handle – cat tries to flee from handling and may growl, hiss, try to strike with the paws, or bite whenever the surgeon approaches, handling is achieved only with vigorous physical restraint and/or protection. 5: impossible to handle – cat becomes very aggressive towards the surgeon and makes containment or handling impossible. Owner opinion (subjective): A custom designed set of options were provided to caregivers for scoring the behaviour of their cat compared to previous consultations. No difference / same as usual. Easier to handle / more relaxed. More difficult to handle / more agitated. More difficult to handle / more aggressive.
Main Findings (relevant to PICO question)	Cats exposed to FFPA had significantly lower CSS when compared to placebo (P < 0.01). The FFPA group had a significantly higher number of cats with scores ≤ 3 on the CSS scale (P = 0.02). There were more cats easier to handle in the FFPA group, however the difference was not significant (P = 0.11). Caregivers of cats exposed to FFPA considered their cat ‘easier to handle / more relaxed’ 12/29 (41%) more often than caregivers whose cats were exposed to placebo 1/33 (3.0%), these results were statistically significant (P < 0.01).
Limitations:	Reproductive status of study population not reported. Reason for presentation and breed not reported for the control group cats 25/87 (29%). No evidence of statistical power and sample size calculation. Composition of the alcohol-based spray used for placebo undefined. Distance of the nozzle for FFPA sprays not defined. Handling techniques not defined but handler consistent for all examinations. Median age of treatment group was significantly lower than control and placebo groups. Unvalidated methods for assessing ease of handling employed. Contributions to study design and writing provided by Ceva Santé Animale (Feliway™ manufacturer), which was declared in the conflict of interest statement. Lack of significant results in relation to ease of handling may have been due to the following limitations: Each score of the scale included variable levels of handling throughout the consultation. When caregivers noted a difference in behaviour, the investigator selected ‘easier to handle / more relaxed’, which might not have been appropriate for both aspects of the question. Typically, a cat that freezes will be easier to handle, despite being stressed, which might have minimised differences assessed through the scale. Assessing the animal’s health based only on physical examination might have caused the investigator to overlook certain pains exacerbated by palpation, causing animals to be more reluctant to handling owing to discomfort. Temperament of animals was not considered.

**Van Vertloo et al. (2021):** 

Population:	Recruitment: Feline participants attended the Lloyd Veterinary Medical Centre of Iowa State University (ISU), USA, during normal business hours from 13:00 to 17:00 in the summers of 2016 and 2017. Cats were recruited from faculty, staff, and students of ISU. Criteria for eligibility and inclusion: At least 1 year of age. Healthy with no diagnosed chronic illnesses. Owned, informed consent and authorisation obtained from caregiver. Criteria for exclusion and rejection: Administered regular medication with the potential to influence blood pressure. Incomplete data: all four consultations not attended. Extreme resistance to handling during data collection. Enrolled study population: 17 castrated males and 22 spayed females. Median age 4 years, ages ranging from 1–15 years. Domestic short hair 34/39 (88%), domestic long hair 3/39 (8%), Siamese mix 1/39 (2%), and Persian 1/39 (2%) breed cats. Median weight 5.9 kg, weights ranging from 3–8.4 kg.
Sample size:	39 cats.
Intervention details:	Group allocation: Randomised (simple randomisation by random number generator) All cats received all treatments and treatment order was randomised. As such, each cat visited the facility four times, however the timing of these visits was not specified. Blinding: Single-blinded. Vocalisation investigator and caregivers were aware of group allocation. Blood pressure investigator was unaware of group allocation. Waiting locations: Waiting room (WR+): a 10 minute period dwelling within the designated hospital waiting room, after which the client and patient were escorted to the examination room, the carrier was placed on a chair with the carrier door facing outwards to the waiting area. Bypassed waiting room (WR–): a 10 minute period dwelling within the examination room. In the examination room, the carrier was placed on a chair with the door facing outwards toward interior of the room. Interventions: F3 feline facial pheromone analogue (FFPA) (Feliway Classic™) exposure (FFPA+): consultation location procedure followed with the addition of a large beach towel sprayed with FFPA placed over the carrier during dwell time. FFPA control (FFPA–): consultation location procedure followed with the addition of an identical towel without FFPA spray in 2016, and no towel or spray in 2017. Consultation procedure: Cats arrived and remained within carriers provided by their caregiver. If a caregiver arrived much earlier than scheduled, a neutral holding place (a closed, quiet office with no human or animal traffic) was provided before the participant was moved to the intended waiting location. During the 10 minute dwelling period, caregivers completed a brief patient medical history. Following the dwell period, the carrier was placed on the examination table, and an additional 10 minutes was provided for acclimation before the exam. Vocalisations performed by the cat during the acclimation period were recorded by one investigator. A second investigator, the clinician, entered the room, removed the cat from the carrier and began the clinical examination – systolic blood pressure was recorded first, followed by heart rate, respiratory rate, body condition score, and body weight. Cuff sizes and position, restraint techniques, carrier type, and body position for blood pressure measurements were noted and kept consistent for subsequent appointments.
Study design:	Prospective, single-blinded, randomised, controlled clinical trial.
Outcome studied:	Vocalisations during acclimation (objective): The number of vocalisations performed in the acclimation period before consultation. Systolic blood pressure (objective): Recorded by vascular Doppler method with headphones. Tail base or rear leg was measured to determine cuff size (40% circumference). Tail base was the preferred site, the rear leg was used when the tail was too short or manipulation not tolerated. Site chosen was recorded and used consistently across all four visits. Hair was clipped on the first visit to facilitate identification of the pulse prior to placement of the Doppler probe and was re-clipped as needed during subsequent visits. Six readings were obtained and the first was excluded. Mean systolic blood pressure was calculated from the remaining values and categorised according to clinical relevance. Normotensive: < 150 mmHg. Borderline hypertensive/hypertensive: 150–179 mmHg. Severely hypertensive: ≥ 180 mmHg. Time to complete blood pressure (objective): Collected from video recordings during 2016 and manually recorded in 2017. Recorded in minutes. Heart rate (objective): Auscultation with a stethoscope, number of beats counted over 15 seconds and multiplied by four. Respiratory rate (objective): Thoracic excursions observed, number of complete breaths counted over 15 seconds and multiplied by four.
Main Findings (relevant to PICO question)	Differences in blood pressure, time to obtain blood pressure measurements, heart rate, and respiratory rate were not significantly associated with FFPA (P > 0.05). Fewer vocalisations were significantly associated with FFPA (P < 0.01), vocalisations were roughly three times greater when cats did not bypass the waiting room and were not provided with FFPA.
Limitations:	History of aggressive behaviour undefined in exclusion criteria. No placebo solution used. Distance of the nozzle and number of FFPA pumps applied not defined. Vocalisations were only recorded during the waiting period and were not recorded during the physical exam or blood pressure measurement. There was no discrimination between the type of vocalisation performed, and whether they were indeed a result of stressed behaviour. Waiting room environment per patient was not recorded – depending on hospital caseload (time of day, day of the week), the waiting room environment can vary greatly, which may have influenced WR+ behaviour. Cat population enrolled may have been more resilient to stressors associated with veterinary visits as they were primarily owned by ISU students and staff, many of whom had participated in other studies. Towel was not placed over carriers in FFPA– treatment in 2017, which may have resulted in differences in stress during the waiting period. Process of removing cats from carriers was not detailed. Headphones were not used consistently with Doppler measurements due to equipment failure. Handlers differed between each year of data collection, minimal restraint was intended however scruffing was used more frequently in 2017.

## Appraisal, application and reflection

During stressful situations, environmental demands exceed the natural regulatory capacity of an organism, eliciting adaptive physiologic and behavioural responses to restore homeostasis (Amat et al., 2016; Lefman & Prittie, 2019; and Stella & Croney, 2019). In cats, physiologic components of this response may include hypertension, tachycardia, tachypnoea, mydriasis, hyperthermia; and biochemical changes such as hyperglycaemia, stress leukogram, and elevated plasma cortisol (Lefman & Prittie, 2019; Quimby et al., 2011; and Tateo et al., 2021). Behavioural changes vary between individuals and may include changes in normal behaviours such as eating, grooming, playing, exploration, urination, defaecation, vocalisation, and affiliation; as well as hiding, vigilance, compulsive, and aggressive behaviours (Amat et al., 2016; Shu & Gu, 2021; and Tateo et al., 2021).

In all five papers, the primary outcome investigated was the response of participants to acutely stressful events within clinical veterinary practice, which was quantified through both objective and subjective parameters (Argüelles et al., 2021; Conti et al., 2017; Kronen et al., 2006; Pereira et al., 2016; and Van Vertloo et al., 2021). Objective measurements included cardiac rate, respiratory rate, systolic blood pressure, electrocardiographic parameters, vocalisations, time to sedation, time to complete blood pressure measurements, cortisol levels, and induction dose of injectable anaesthetic (Argüelles et al., 2021; Conti et al., 2017; and Van Vertloo et al., 2021). One study used only objective measurements (Van Vertloo et al., 2021). Subjective assessments included behavioural descriptions applied to numerical rating scales (Argüelles et al., 2021; Conti et al., 2017; Kronen et al., 2006; and Pereira et al., 2016). Two studies used only subjective measurements in their study design (Kronen et al., 2006; and Pereira et al., 2016).

Good subjective stress assessment tools should select for species-specific behavioural measures that are consistent across repeated testing (Urrutia et al., 2019). Stress scales are commonly developed for this purpose and can be used to identify stress and assess its severity (Lefman & Prittie, 2019). Scales to assess feline behaviour have been developed, however the primary focus within the literature has been the shelter environment, and therefore chronic stress (Kessler & Turner, 1997; and Hirsch et al., 2021). Additionally, the scales that do exist, such as the Cat Stress Score, are challenging to validate (Hirsch et al., 2021; and Urrutia et al., 2019). Consequently, all studies evaluated used unvalidated behavioural scales to assess acute stress. Limitations associated with unvalidated behavioural scales include insufficient detail, which limits the refinement of a scale, and inability to capture the complexity of feline stress responses (de Rivera et al., 2017; Pereira et al., 2016). For example, certain stressed cats may freeze and thus be interpreted as easy to handle, while others may be more active, which can be interpreted as less fearful (Carlstead et al., 1993; Kronen et al., 2006; and Pereira et al., 2016). Additionally, the effect of some factors, such as vocalisation, have produced conflicting results within the literature (de Rivera et al., 2017; Urrutia et al., 2019; and Van Vertloo et al., 2021). A laboratory based model utilising open-field testing and the human interaction test  has been developed for assessing acute fear and anxiety in cats (de Rivera et al., 2017). This could be employed in a laboratory based study upon the influence of FFPA on acute stress. Ultimately, a validated acute stress scale for use within a clinical veterinary context would be of use.

The findings of each study must be interpreted in context with the inherent difficulties associated with accurate stress measurement and assessment. Additionally, individual responses to the same stressor vary, and interpretation is further complicated by the numerous confounding factors that may influence how, and to what degree, stress is expressed through behaviour, including health status, temperament, and age (Lefman & Prittie, 2019).

Normal animal behaviour may be altered by the presence of pain, illness, and disease (Mills et al., 2020; Lefman & Prittie, 2019; Riemer et al., 2021; and Waran et al., 2007). All studies screened participants on the basis of health and excluded animals previously diagnosed with disease or taking medications (Argüelles et al., 2021; Conti et al., 2017; Kronen et al., 2006; Pereira et al., 2016; and Van Vertloo et al., 2021). However, Pereira et al. (2016) only utilised physical examination without palpation during this process, and thus may not have eliminated pain as a confounding factor.

In cats, temperament has been implicated as a factor that may influence the behavioural expression of stress (Amat et al., 2016; Foster & Ijichi, 2017; and Stella & Croney, 2019). Temperament is defined as the behavioural differences between individuals that are stable across time and contexts (Amat et al., 2016; Travnik et al., 2020; and Travnik & Sant’Anna, 2021). Coping styles are a facet of temperament related to the physiologic and behavioural stress response of an animal, which are consistent over time (Koolhaas et al., 1999). Two coping styles, known as proactive and reactive, have been described in the domestic cat (Stella & Croney, 2019; and Travnik et al., 2020). The proactive coping style is physiologically associated with high sympathetic nervous system activation and low hypothalamic-pituitary-adrenal (HPA) axis activation (Stella & Croney, 2019). Behavioural responses involve offensive aggression and territorial control (Koolhaas et al., 1999; Stella & Croney, 2019; and Travnik et al., 2020). Conversely, the reactive coping style is physiologically characterised by high parasympathetic nervous system activation and higher HPA-axis activation (Stella & Croney, 2019). Behaviourally this strategy involves withdrawal, immobility, and low levels of aggression (Koolhaas et al., 1999; Stella & Croney, 2019; and Travnik et al., 2020).

Two of the examined studies, Kronen et al. (2006) and Van Vertloo et al. (2021), excluded participants on the basis of aggressive behaviour. It is possible therefore that participants with proactive coping styles, which behaviourally align with offensive aggression, were preferentially excluded. This may have skewed findings associated with subjective behavioural assessment, particularly in relation to activity levels. The confounding effect of temperament between selected participants is likely to have been negated by the study design employed by both Conti et al. (2017) and Van Vertloo et al. (2021) as all study participants received all treatments in a randomised fashion. The Feline Temperament Score offers a validated, non-invasive means of objectively quantifying feline temperament for inclusion in statistical analysis (Foster & Ijichi, 2017; and Siegford et al, 2003). In future study designs where all participants do not receive all treatments, the Feline Temperament Score may be an appropriate means of mitigating temperament as a confounding factor.

As animals grow and mature, their behaviour changes and consequently, behavioural expressions of stress in cats are likely to evolve over time (Foster & Ijichi, 2017; Tateo et al., 2021; and Urrutia et al., 2019). Pereira et al. (2016) and Van Vertloo et al. (2021) actively excluded participants on the basis of age and only accepted cats older than 26 weeks and 1 year of age, respectively. One rationale for which was to prevent kitten behavioural reactions (Pereira et al., 2016), however no rationale for this exclusion was provided by Van Vetrloo et al. (2021). Studies designed by Argüelles et al. (2021) and Kronen et al. (2006) attempted to control this confounding factor by demonstrating no significant difference in age between treatment groups. Van Vertloo et al. (2021) controlled the confounder using 2x2 factorial design where all cats received all treatments and paired analysis was performed. Additionally, age was included in statistical analysis as a covariate, however no significant difference was found (Van Vertloo et al., 2021). In comparison, median age differed between treatment groups in Pereira et al. (2016) as the median age of the FFPA treatment group was significantly lower. Conti et al. (2017) failed to demonstrate within statistical analysis the presence or absence of significant age difference. Groups in this study were randomised, which reduces the likelihood of confounding bias, however it should be noted that the method of randomisation was not defined.

Regarding randomisation, one reviewed study, by Argüelles et al. (2021), did not employ randomisation to assign treatment groups. This was due to the method of study recruitment, where caregivers were offered the choice to follow a transport protocol when bringing their cat to the clinic for surgery, and therefore enrol their cat in the treatment group (Argüelles et al., 2021). Cats who had been exposed to the protocol, in which the protocol had been applied correctly on the day of surgery, were enrolled in the treatment group (Argüelles et al., 2021). This method of enrolment is likely to have introduced study bias, either through factors influencing caregiver compliance with the low-stress transport protocol such as practical viability, attitudes towards cat welfare, caregiver demographics, or unknown factors. These were not investigated. Conversely, all other studies employed some means of randomisation (Conti et al., 2017; Kronen et al., 2006; Pereira et al., 2016; and Van Vertloo et al., 2021). However, methods used were only reported by Kronen et al. (2006), Pereira et al. (2016), and Van Vertloo et al. (2021), who used block randomisation by lottery, simple randomisation based upon order of arrival at the clinic, and simple randomisation based upon random number generator, respectively.

Sample size power calculations were performed by Conti et al. (2017) and Van Vertloo et al. (2021) using data from previous studies, however the mean and standard deviations utilised were not reported. Five individual data sets may have been used by Conti et al. (2017) (Hanås et al., 2009; Lin et al., 2006; Sparkes et al., 1999). Similarly, three data sets were available within the resource used by Van Vertloo et al. (2021) (Sparkes et al., 1999). Consequently, values for both sample size calculations cannot be externally verified, and post-hoc power analysis is typically discouraged (Zhang et al., 2019). Additionally, Argüelles et al. (2021), Kronen et al. (2006) and Pereira et al. (2016) showed no evidence of sample size power calculation and therefore the suitability of their sample sizes cannot be verified.

Behavioural assessment is subjective and interpretations will vary between operators (Wemelsfelder, 1997). Additionally, operator training will influence the ability to recognise and interpret behaviours successfully (Wemelsfelder, 1997). A single-blinded operator was used by Conti et al. (2017) and Pereira et al. (2016), reducing the effect of inter-operator variability. In contrast, Argüelles et al. (2021) used two, Kronen et al. (2006) used five, and Van Vertloo et al. (2021) used an unspecified number of assessors. While none of the studies claimed to have employed an operator specialised in feline ethology, Conti et al. (2017), Kronen et al. (2006), and Pereira et al. (2016) ensured operators were trained for study specific investigations. Although there are difficulties associated with producing accurate and reliable subjective behavioural measurements to quantify stress, observation of behaviour is an important aspect of stress assessment (Lefman & Prittie, 2019).

In terms of study blinding, Argüelles et al. (2021), Kronen et al. (2006), Pereira et al. (2016) and Van Vertloo et al. (2021) included complete descriptions of study blinding, while Conti et al. (2017) did not. Van Vertloo et al. (2021) did not blind operators collecting vocalisation data for practical reasons. This is acceptable however, as the outcome was an objective parameter (Day & Altman, 2000; and Giuffrida et al., 2012). It should also be noted that if placebo composition resulted in concealment of allocation, the effect of blinding upon operators would be nullified (Day & Altman, 2000 Schulz, 2005; and Schulz & Grimes, 2002).

Three of the evaluated studies incorporated a placebo. Conti et al. (2017) used an ethanol 70% spray, Pereira et al. (2016) used an alcohol-based spray, and Kronen et al. (2006) used Feliway Classic™ carrier medium, which according to manufacturers is approximately 90% ethanol (Ceva Animal Health, 2022a). In these situations, placebos may have been distinguishable through either appearance or scent. Kronen et al. (2006) stipulated that identical bottles were used while Pereira et al. (2016) utilised two colourless liquid solutions, visual difference between interventions was not reported by Conti et al. (2017). Humans are unable to detect FFPA by smell (Ceva Animal Health, 2022b; and DePorter, 2016). However, in situations where the composition of placebo differed from the FFPA carrier medium solution, a slight difference in smell may have been discernable. This may have been the case when ethanol 70% was employed by Conti et al. (2017) and an alcohol-based spray of unknown composition by Pereira et al. (2016).

The pheromone product used in all studies was a synthetic F3 fraction of FFPA marketed as Feliway Classic™ with active constituents 100 mg/mL synthetic FFPA and 89.2% ethanol (Argüelles et al., 2021; Ceva Animal Health, 2022a; Conti et al., 2017; Kronen et al., 2006; Pereira et al., 2016; and Van Vertloo et al., 2021). UK manufacturer’s instructions state that 8-10 pumps should be applied 15 minutes before the introduction of a cat (Ceva Animal Health, 2022b). A single pump is defined as one depression of the nozzle, with the bottle held vertically (Ceva Animal Health, 2022a). The nozzle is intended to be held at a distance of approximately 10 cm from the intended spray site, and 20 cm above the ground (Ceva Animal Health, 2022a). It should be noted that instructions vary by region. Three to four pumps are recommended in Australia (Ceva Animal Health, 2022c), and a period of 10 minutes is recommended in the USA (Ceva Animal Health, 2021).

In the studies reviewed, a variety of methods were used to apply FFPA. Argüelles et al. (2021) applied four pumps to the carrier, and three to the car 30 minutes before introducing a cat. Distance of the nozzle was not defined, and FFPA diffuser was also used in the consultation room (Argüelles et al., 2021). Conti et al. (2017) applied FFPA throughout the location but did not define a number of pumps. Pumps were applied 10 cm from objects and 20 cm from the floor. A duration of 15 minutes was used before introducing a cat (Conti et al., 2017). Kronen et al. (2006) applied an undefined number of pumps 10 cm from floor 1–1.5 minutes before introducing a cat. A pump was defined as depression of the nozzle for 1–2 seconds (Kronen et al., 2006). Pereira et al. (2016) applied five pumps 15 minutes before exposure at an undefined distance. Van Vertloo et al. (2021) sprayed an undefined number of pumps at an undefined distance onto a towel, which was immediately placed over cat carriers. It is unclear whether cats may have been able to hear application of the spray by Van Vertloo et al. (2021). The spritzing sound may be associated with the sound of hissing by some cats and can cause stress (DePorter, 2016).

This variation in application may have confounded results for a number of reasons. Firstly, time before FFPA exposure is intended to allow scent of the ethanol carrier medium to dissipate (Conti et al., 2017; DePorter, 2016; and Pereira et al., 2016). Cats as a species have a very sensitive sense of smell and it is advised that wherever possible, interference with feline olfactory signals and scent profiles is avoided (Ellis et al., 2013; and Herron & Shreyer, 2014). Insufficient time before exposure may have exposed participants to a pungent alcohol scent, which may have influenced behaviour and confounded results (DePorter, 2016). Secondly, the time of exposure to FFPA before exposure to stressful stimuli was only recorded by Conti et al. (2017), where the environment remained consistent with the addition of FFPA for 10 minutes before veterinary manipulations. Thirdly, the amount of FFPA product administered is likely to have varied between studies. No studies reported the type of spray bottle utilised and whether the bottle design was consistent with commercially available products. Variation in bottle design in terms of shape and nozzle configuration, along with variation in the number of pumps of FFPA applied complicates comparison of results. Finally, no studies reported upon FFPA product storage. Manufacturers recommend that FFPA products be shaken before use and stored below 25 °C in a well-ventilated area with containers tightly closed (Ceva Animal Health, 2022a). Failure to adhere to these guidelines may have led to product degradation and decreased exposure to FFPA when compared to reported levels.

Across the studies, handling methods also varied considerably. Argüelles et al. (2021) employed low-stress protocols at all times and techniques were defined as following the 2011 American Association of Feline Practitioners (AAFP) and International Society of Feline Medicine (ISFM) Feline Handling Guidelines (Rodan et al., 2011). Van Vertloo et al. (2021) ensured all handlers were trained by a certified veterinarian in low-stress handling techniques as defined by Yin (2009). Restraint techniques used during the study were also reported and while minimal restraint was preferred and used where possible, cats were scruffed (Van Vertloo et al., 2021). Handling techniques were not specifically defined by Conti et al. (2017), Kronen et al. (2006), and Pereira et al. (2016) however Conti et al. (2017) stated cats were evaluated from least to most stressful manipulation. Reporting of handling methods influences reproducibility and interpretation of results, particularly in regard to the additive effect of stress. 

To minimise handling as a confounding factor, consistency across study participants is imperative. Pereira et al. (2016) ensured all consultations were performed by the same handler. Two handlers were employed by Argüelles et al. (2021) and Conti et al. (2017), the latter of which consistently used the same order of examination. Kronen et al. (2006) employed an undefined number of handlers, some of whom were final-year veterinary students and as such, handling expertise is likely to have varied. Handling techniques of Van Vertloo et al. (2021) varied considerably in terms of scruffing, clipping, headphone use, and towel use. One participant at a single visit was scruffed in 2016 (1/88 (1%), while at 14/68 (21%) of visits participants were scruffed in 2017 when different handlers were employed. Patients were also clipped when required, and thus variations in coat length and thickness may have increased frequency of clipping for some participants across visits. Due to equipment failure, headphone use for Doppler blood pressure measurements was also inconsistent and used more frequently in 2016 than 2017. Finally, towels were placed over carriers in the FFPA treatment group in 2016 but not 2017. These examples of variation in handling throughout studies may have confounded results.

When investigating the effect of FFPA upon acute stress, it is possible that the additive effect of stress associated with the environment coupled with data collection methods may have overcome the protective influence of FFPA and minimised the significance of findings (Argüelles et al., 2021; Van Vertloo et al., 2021). Each study investigated the effect of acute stress, which for the purposes of this review was defined as a stressor lasting a duration of one hour or less (Dickerson & Kemeny, 2004). However, stress is recognised to have an additive effect where animals exposed to several stressors experience a greater stress response than when exposed to a single stressor (Amat et al, 2016). Acute stress within a veterinary context is likely to be multifactorial in nature, and this may have influenced the degree of subject response (Amat et al, 2016; Shu & Gu, 2021; Stella & Croney, 2019).

No adverse effects due to FFPA application were reported in any of the reviewed studies (Argüelles et al., 2021; Conti et al., 2017; Kronen et al., 2006; Pereira et al., 2016; and Van Vertloo et al., 2021). Additionally, other investigations into feline pheromonotherapy report that FFPA appears free of side effects and psychopharmacological contraindications (Mills, 2005).

The clinical application of pheromonotherapy may allow clinicians to disseminate positive messages throughout a cat’s environment, influencing their emotional and behavioural responses (DePorter, 2016; Mills, 2005). Formulation for clinical application is an important consideration. The studies investigated predominantly used spray formulations (Conti et al., 2017; Kronen et al., 2006; Pereira et al., 2016; and Van Vertloo et al., 2021), however one study also utilised a diffuser (Argüelles et al., 2021). Spray formulations are portable and allow specific locations to be targeted with pheromone on an as-needed basis (Ceva Animal Health, 2022b; DePorter, 2016). Their primary disadvantage however is logistical in that they must be applied prior to exposure and reapplied often to provide a continuous effect (Ceva Animal Health, 2022b; DePorter, 2016). In situations where pheromone exposure is required in a specific location, electronic diffusers may be more practical (DePorter, 2016). Besides the logistical advantages, diffusers also provide greater preventative protection against acute stress as FFPA provides a greater protective effect if provided while cats are in a lowered state of arousal (DePorter, 2016). Current best practice implementation of pheromonotherapy in clinical veterinary practice for the mitigation of acute stress includes the use of diffusers placed within rooms frequented by feline patients, along with FFPA spray applied to towels and bedding 15 minutes prior to handling or confinement (DePorter, 2016).

Clinical application must also acknowledge the limitations of pheromonotherapy. Pheromone products are designed to influence emotional responses rather than completely control behaviours (DePorter, 2016). Consequently, FFPA should not be used alone as an exclusive measure for stress mitigation (Conti et al., 2017; DePorter, 2016). A best practice clinical approach requires minimisation of environmental stressors through feline friendly clinic design and handling (Conti et al., 2017; Ellis et al., 2013; Herron & Shreyer, 2014; and Yin, 2009).

In conclusion, four of the five studies reviewed found significant improvement in some indicators of acute stress following FFPA exposure. Pereira et al. (2016) showed that FFPA reduced patient stress during routine physical examination, and improved caregiver impression of patient relaxation and ease of handling. Van Vertloo et al. (2021) revealed FFPA decreased vocalisations but had no effect upon systolic blood pressure during physical examination. Argüelles et al. (2021) demonstrated reduced time to sedation and propofol induction dose for routine surgical procedures when a transport protocol incorporating FFPA was applied. Finally, Kronen et al. (2006) determined FFPA calmed but did not reduce struggling during venous catheterisation. Conti et al. (2017) found no significant effect of FFPA upon behavioural or physiologic measures of acute stress during physical examination. Singularly, each finding is not profound however when combined, these findings suggest that the application of FFPA within a clinical veterinary context may provide some benefit toward the mitigation of acute stress in cats.

All studies reviewed were weakened to a degree by various limitations. Consequently, a further study using a calculated sample size of healthy, temperament tested patients, FFPA applied according to manufacturer’s instructions, and a validated stress scale designed for application during acute stress would be beneficial. Additionally, as FFPA diffusers have been identified as the most practical method of application during acutely stressful events, inclusion of diffusers within the study design would be valuable.

## Methodology

**Search strategy:** 

Databases searched and dates covered:	CAB Abstracts via Web of Science: 1910–2023 PubMed: 1966–2023 Scopus: 1960–2023
Search strategy:	CAB Abstracts: cat OR cats OR feline* OR felid* (Topic) and vet OR veterinary OR veterinarian OR clinic* OR hospital* OR practice* OR consult* (Topic) and feliway OR pheromone OR "facial pheromone" OR "F3 pheromone" OR "F3 analogue" (Topic) and fear* OR anxiety OR anxieties OR anxious* OR stress* OR phobi* OR panic* OR behavio?r (Topic) PubMed: (((cat OR cats OR feline* OR felid*) AND (vet OR veterinary OR veterinarian OR clinic* OR hospital* OR practice* OR consult*)) AND (feliway OR pheromone OR "facial pheromone" OR "F3 pheromone" OR "F3 analogue")) AND (fear* OR anxiety OR anxieties OR anxious* OR stress* OR phobi* OR panic* OR behavio?r) Scopus: (TITLE-ABS-KEY(cat OR cats OR feline* OR felid*) AND TITLE-ABS-KEY(vet OR veterinary OR veterinarian OR clinic* OR hospital* OR practice* OR consult*) AND TITLE-ABS-KEY(feliway OR pheromone OR “facial pheromone” OR “f3 pheromone” OR “f3 analogue”) AND TITLE-ABS-KEY(fear* OR anxiety OR anxieties OR anxious* OR stress* OR phobi* OR panic* OR behavio?r))
Dates searches performed:	03 Aug 2023

**Exclusion / inclusion criteria:** 

Exclusion:	Publications older than 30 years. Non-English language studies. Non-experimental design: case studies, systematic reviews, book chapters, conference proceedings. Unrelated to PICO parameters: non-feline participants, non-F3 feline facial pheromonal agents, study not measuring outcomes associated with acute stress, study participants not within a clinical veterinary context, intervention not compared to a control measure.
Inclusion:	Experimental studies: controlled trials. Investigating the effect of F3 feline facial pheromone analogue applied before acutely stressful events within a clinical veterinary context. To differentiate between papers investigating chronic stress, acute stressors were defined as lasting 1 hour or less.

**Search outcome:** 

Database	Number of results	Excluded – > 30 years old	Excluded – Non-English language publication	Excluded – Non-experimental design	Excluded – Not related to PICO question	Total relevant papers
CAB Abstracts	98	5	8	69	11	5
PubMed	22	0	0	11	7	4
Scopus	48	0	4	31	8	5
Total relevant papers when duplicates removed						5

## ORCiD

Ebony Crump: https://orcid.org/0000-0001-6771-6511

## Conflict of interest

The author declares no conflicts of interest.

## Disclaimer

Knowledge Summaries are a peer-reviewed article type which aims to answer a clinical question based on the best available current evidence. It does not override the responsibility of the practitioner. Informed decisions should be made by considering such factors as individual clinical expertise and judgement along with patient’s circumstances and owners’ values. Knowledge Summaries are a resource to help inform and any opinions expressed within the Knowledge Summaries are the author’s own and do not necessarily reflect the view of the RCVS Knowledge. Authors are responsible for the accuracy of the content. While the Editor and Publisher believe that all content herein are in accord with current recommendations and practice at the time of publication, they accept no legal responsibility for any errors or omissions, and make no warranty, express or implied, with respect to material contained within. For further information please refer to our Terms of Use.
